# Arsenic metabolism in high altitude modern stromatolites revealed by metagenomic analysis

**DOI:** 10.1038/s41598-017-00896-0

**Published:** 2017-04-21

**Authors:** Daniel Kurth, Ariel Amadio, Omar F. Ordoñez, Virginia H. Albarracín, Wolfgang Gärtner, María E. Farías

**Affiliations:** 1 Planta Piloto de Procesos Industriales y Microbiológicos (PROIMI), CCT Tucumán, CONICET, San Miguel de Tucumán, Argentina; 2 E.E.A. Rafaela, Instituto Nacional de Tecnología Agropecuaria (INTA), CCT Santa Fe, CONICET, Rafaela, Argentina; 3 Facultad de Ciencias Naturales e Instituto Miguel Lillo, Universidad Nacional de Tucumán, San Miguel de Tucumán, Argentina; 4 Max-Planck Institute for Chemical Energy Conversion, Mülheim an der Ruhr, Germany

## Abstract

Modern stromatolites thrive only in selected locations in the world. Socompa Lake, located in the Andean plateau at 3570 masl, is one of the numerous extreme Andean microbial ecosystems described over recent years. Extreme environmental conditions include hypersalinity, high UV incidence, and high arsenic content, among others. After Socompa’s stromatolite microbial communities were analysed by metagenomic DNA sequencing, taxonomic classification showed dominance of Proteobacteria, Bacteroidetes and Firmicutes, and a remarkably high number of unclassified sequences. A functional analysis indicated that carbon fixation might occur not only by the Calvin-Benson cycle, but also through alternative pathways such as the reverse TCA cycle, and the reductive acetyl-CoA pathway. Deltaproteobacteria were involved both in sulfate reduction and nitrogen fixation. Significant differences were found when comparing the Socompa stromatolite metagenome to the Shark Bay (Australia) smooth mat metagenome: namely, those involving stress related processes, particularly, arsenic resistance. An in-depth analysis revealed a surprisingly diverse metabolism comprising all known types of As resistance and energy generating pathways. While the *ars* operon was the main mechanism, an important abundance of *arsM* genes was observed in selected phyla. The data resulting from this work will prove a cornerstone for further studies on this rare microbial community.

## Introduction

Arsenic is not only a natural component in the Earth crust and in various minerals, but it is also a major contaminant of aquatic ecosystems worldwide^1^. It is a widespread element and its main sources are natural, mostly associated to volcanic regions and hydrothermal vents. In nature, microorganisms cope with arsenic toxicity in different ways: extracellular precipitation, chelation, intracellular sequestration, active extrusion from the cell or biochemical transformation (by redox processes or methylation). Some microorganisms are also able to utilize this metalloid as a metabolic energy source through either arsenite oxidation or arsenate reduction. A number of genes are involved in such processes, and the marker genes representative of each of them can be selected to provide an overview of the arsenic biogeochemical cycle, namely *arsABCDH*, *acr3*, *arsM*, and those related to energetic metabolism: *aioA* and *arrA* ^2^.

Among environments with high arsenic content, High Altitude Andean Lakes (HAAL) comprise a system of shallow waters that are distributed across the Puna (high plateau) at altitudes that vary between 3,000 m and 6,000 m above sea level (masl)^3^. HAAL lie within the Central Andean volcanic zone (14°–28°S), one of the regions in the world most affected by explosive volcanism over the past several million years and where some of the largest supervolcanoes in the world^4^ can be found. Microbial communities with different levels of complexity that thrive at HAAL include biofilms, microbial mats, evaporites, and microbialites^5–8^. In these environments high concentrations of arsenic are found naturally in the water^9–15^. In selected locations, its concentration is extremely high (up to 119 mg L^−1^) as in the case of Diamante Lake in the caldera of Cerro Galán Volcano in Catamarca, Argentina. At the mentioned site, archaeal biofilms are capable of oxidizing As(III) and reducing As(V), a process that couples with energy generation, which was the first demonstration of the presence of these metabolisms in Haloarchaea^8^. Arsenic biogeochemical cycling was also shown to occur in other HAAL locations in Chile like Salar de Ascotán^16^, where some of the organisms involved were initially identified by enrichment cultures^17^. Later on, the genes involved in arsenic metabolism were prospected in a number of sites with arsenic concentrations spanning six orders of magnitude^18^. High As content was also detected at Socompa Lake which is located in the desert region of the province of Salta, Argentina, at the base of the Socompa Volcano at 3,570 masl, where modern stromatolites developed under the pressure of extreme environmental factors similar to the ones present in Early Earth’s atmosphere^19^. The bacteria isolated from these stromatolites are, in fact, extremophiles able to resist severe stress conditions, including UV radiation, heavy metals, salinity and, most interestingly, arsenic. Previous works have highlighted the importance of arsenic at HAAL^12^, with resistant strains isolated from the Socompa stromatolites^20–22^. HAAL´s arsenic resistance mechanisms involved *ars* operons including Acr3 type efflux pumps. Though it was contended at first that the presence of multiple pumps could have been the cause behind enhanced resistance in *Exiguobacterium* sp. S17, Acr3-type pumps had not been previously reported in this genus^22^.

Study, characterization and description of the stromatolites are of utmost interest since they are considered to be the earliest evidence of life on Earth, with geological records dating back 3.5 billion years^23–25^. Other modern stromatolites have been discovered in the world, located at low to medium altitudes where microorganisms cope with different stress conditions. Some of the most thoroughly studied systems include the hypersaline region of Hamelin Pool, Shark Bay in Western Australia^26^, and the shallow subtidal regions at the margin of Exuma Sound in the Bahamas^27^. In recent years, microbial mat communities with different degrees of lithification at HAAL have been characterized and proposed as Early Earth models^5–7, 19, 28–30^. According to recent findings^31^, an exceptional characteristic of ancient stromatolite systems might have been their arsenic metabolism. These authors propose that arsenic cycling might likely have been an energy source in a Precambrian lacustrine stromatolite. However, in modern sea level systems arsenic is present in only minute amounts, which is another notable difference when compared to the high concentrations found at Socompa and other Andean systems.

Based on the evidence exposed above, As seems to be an important component in the Andean stromatolite geochemistry and a detailed study of the role of the As cycle in modern stromatolites is an important issue. To this end, the present work provides a metagenomic perspective of high altitude stromatolites, with special focus on the systems involved in arsenic metabolism, and their role in biogeochemical cycling and energy generation.

## Results and Discussion

### Description of the site

Socompa Lake is a HAAL located in the desert area of the province of Salta in the Argentine Puna region, at the base of the Socompa Volcano at 3,570 masl^19, 28^. Extreme environmental conditions in this site include hypersalinity, high thermal amplitude with daily temperatures that range from −10 °C to 20 °C in summer and −20 °C to 10 °C in winter, UV solar irradiance that reaches 68 W m^−2^ ^19, 32^, low O_2_ pressure, low nutrient availability and, primarily, high arsenic content (18.5 mg L^−1^). The volcano is deemed still active, with the last eruption estimated to have occurred less than 10,000 years BP. The ash deposits in the entire region have been extremely well preserved over long time spans due to the hyper-arid conditions which have prevailed over millions of years^4^. A prolonged exposure of ash to weathering may instigate the slow release of elements from the structure of constituent mineral phases, transporting potential toxic trace elements such as arsenic and heavy metals to the environment, which –in addition to high evaporation rates and relatively high arsenic concentration in groundwater^33^– is likely to contribute to arsenic accumulation in the lake. The hydrothermal water that goes into the lake is part of the modern Andean volcanic system^34^. It is here that modern Stromatolites were reported and characterized for the first time by Farías and colleagues^19, 28^. These structures were found around the border of Socompa Lake during the summer when they are partially exposed depending on the tide and hydrological regime; on the contrary, they are completely submerged during winter and spring.

### General description of the metagenome

#### Assembly and annotation

DNA from the Socompa stromatolite microbial community was obtained and sequenced by shotgun strategy with Illumina technology. Quality filtering yielded 12.3 Gbp for further analysis. As an initial approach to the data, the reads were analysed with Nonpareil^35^, which provided a 92% estimate for the average coverage in Socompa, suggesting that assembly and binning might be feasible. IDBA_UD^36^ was used for assembly since it can deal with differential coverage of genomes due to different abundances. 391,949 contigs were produced, with a mean length of 954 bp, a N50 of 1359 bp, and a total length of 374,092,121 bp. From the latter, 79,915 contigs with lengths above 1000 bp were kept for further analysis, encompassing 219,061,845 bp of the assembled sequences (Supplementary Table S1). Gene annotation on these contigs predicted 251,312 proteins. Based on the predicted ORF, one gene was found every 1 kb, which is the expected coding density for microbial species. All data were uploaded to the MG-RAST server^37^ for further analysis.

#### Phylogenetic analysis

**Figure 1:**
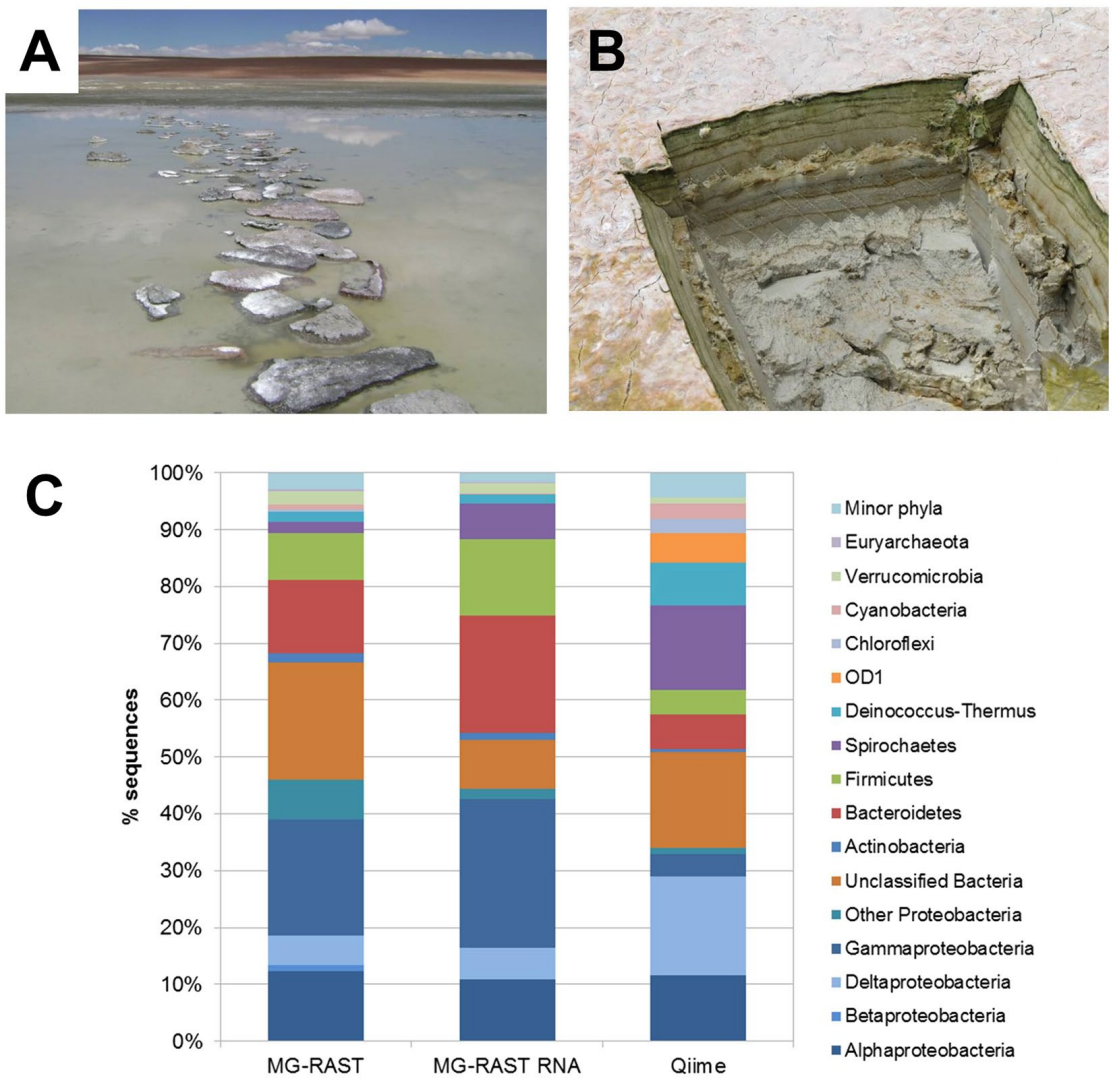
(**A**) Socompa stromatolites. (**B**) Vertical section. (**C**) Taxonomic diversity analysed by different methods: MG-RAST results based on assembled data compared to M5NR database, and MG-RAST assignment of RNA reads, updated Qiime analysis on published 16S rRNA data^19^.

It is known that depending on which method is applied, diversity analysis can be biased. Specifically, the comparison between 16S rRNA gene amplicon data and shotgun metagenomics yields significantly different results^38^. For the Socompa sample, 16S rRNA amplicon and WGS sequencing results are shown in Fig. 1C. After the assembled sequences were analysed in MG-RAST^37^ against the M5NR database, Proteobacteria appeared as the most abundant phylum (46%) including 20% of Gammaproteobacteria. The second most abundant group contained unclassified sequences (20%), followed by other important phyla such as Bacteroidetes (13%) and Firmicutes (8%). This ordering trend is mirrored by the analysis of rRNA metagenomic sequences, classified in the MG-RAST server where, again, Proteobacteria were dominant with 44%, including 26% of Gammaproteobacteria. Bacteroidetes (21%) and Firmicutes (13%) were also well represented. rRNA 16S gene amplicons obtained by Farías and colleagues^19^ (marked as Qiime in Fig. 1C) were analysed with the methods reported as well as updated software and databases, resulting in 34% Proteobacteria, 17% Unclassified, 15% Spirochaetes, and 7.5% Deinococcus-Thermus.

Different methods at the phylum level yield similar general trends, such as the presence of Proteobacteria, Bacteroidetes and Firmicutes in all cases. However, percentages vary and some differences are observed, since certain methods seem to over-represent definite groups: Deinococcus-Thermus and Spirochaetes with Qiime and Gammaproteobacteria with MG-RAST. The presence of Deinococcus-Thermus in this environment is expected given the known UV-resistance of members of the *Deinococcus* genus^39^. However, their relative abundance is difficult to assess precisely because of the different results provided by the methodologies used. PCR amplification and analysis of environmental DNA from the top layer (0–0.3 mm depth) suggest 88% abundance in this layer (unpublished results), while a 7% relative abundance in a 5 cm deep sample appears to be an overestimate. There might be other phylum members at different depths of the stromatolite, but we believe that the high value rendered might be due to 16S rRNA gene PCR bias. The presence of Gammaproteobacteria in the MG-RAST annotation might be influenced by an overrepresentation of these sequences in the M5NR database, over those with fewer or no sequenced isolates, as similarly proposed by Shi *et al*.^40^.

A striking characteristic of this community is the low abundance of Cyanobacteria. Although these could be the primary producers, other abundant organisms such as the anoxygenic photosyntesizer members of Alphaproteobacteria *Roseobacter*, *Rhodobacter*, *Roseovarius* (~1% abundance each) could also be functioning as contributors. Low abundance of Cyanobacteria is not unusual among microbial mats in HAAL communities^5–7, 29^ or other environments with high UV incidence like Shark Bay^41^. The contribution of diatoms is unknown, but they are present and can be seen in electron microscopy images^19^.

#### Functional analysis

Modern microbial mats and microbialite growth is believed to depend on two critical aspects: the “alkalinity engine” and the exopolymeric matrix (EPS)^42^; microbial metabolism can influence both. The balance between microbial activities can determine the net accumulation of carbonate minerals and the lithification of mats^43^. Carbon fixation removes CO_2_ from the environment, and when the rate exceeds CO_2_ replenishment through diffusion, bicarbonate dissociates into CO_2_ and OH^−^, creating alkalinity that favors CaCO_3_ precipitation^42^. This effect is usually observed with oxygenic photosynthesis carried out mostly by Cyanobacteria resulting from the Calvin-Benson cycle for CO_2_ fixation. However, carbon fixation at Socompa might also follow alternative pathways. Analysis of the abundance of key enzymes from each of the known carbon fixation pathways^44^ shows that the most abundant is the ATP–citrate lyase, which represents the Arnon–Buchanan cycle (rTCA), followed by RuBisCO for the Calvin-Benson cycle and the bifunctional enzyme carbon monoxide dehydrogenase/acetyl-CoA synthase for the reductive acetyl-CoA pathway (Wood-Ljungdahl pathway) (Supplementary Table S2). Also present is 4-hydroxybutyryl dehydratase, a key enzyme for the dicarboxylate–hydroxybutyrate and hydroxypropionate–hydroxybutyrate (HP/HB) cycles. Similar results were observed in Shark Bay microbial systems, where a number of alternative carbon fixation pathways were found, including the ancient HP/HB cycle^41^, but the relative contribution of each to the whole carbon fixation and precipitation process remains unknown.

An alternative process contributing to carbon precipitation is sulfate reduction^42^. Sulfate reducers are well represented at Socompa, judging not only from taxonomic data showing Deltaproteobacteria –mostly from the *Desulfobacteraceae* family– but also from the presence of key genes for sulfate reduction, such as sulfate adenyltransferase (*Sat*), and dissimilatory sulfite reductase (*dsrAB*) (Supplementary Table S2). In fact, precipitation by this mechanism is thought to occur in the deeper layers of the stromatolite^19^, where some of these anoxygenic microorganisms could play a major role. The influence of sulfate reducers in precipitation has been observed in other hypersaline microbial mats, such as Salt Pan in Eleuthera, Bahamas^45^.

Besides sulfur and carbon cycling, other relevant metabolic markers are related to the nitrogen cycle (Supplementary Table S2). Though most genes in this cycle seem to be present, there is a significant absence of *amo* genes that code for ammonia monooxygenase – involved in nitrification. Previous analysis using specific primers yielded PCR amplification products only for betaproteobacterial *amo* genes related to the *Nitrosomonas* lineage^19^. Thus, combining PCR and metagenomic data, the nitrogen cycle is complete, but the Betaproteobacteria are present in a very low relative abundance and this might explain why *amo* genes are not detected in metagenomic sequences. Nitrogen fixation is performed mostly by Deltaproteobacteria (Supplementary Fig. S1), in agreement with previous results^19^. Thus, in Socompa, this group might be involved in both sulfate reduction and nitrogen fixation, and could consequently be playing a central metabolic role in this system.

### Comparative analysis

**Figure 2:**
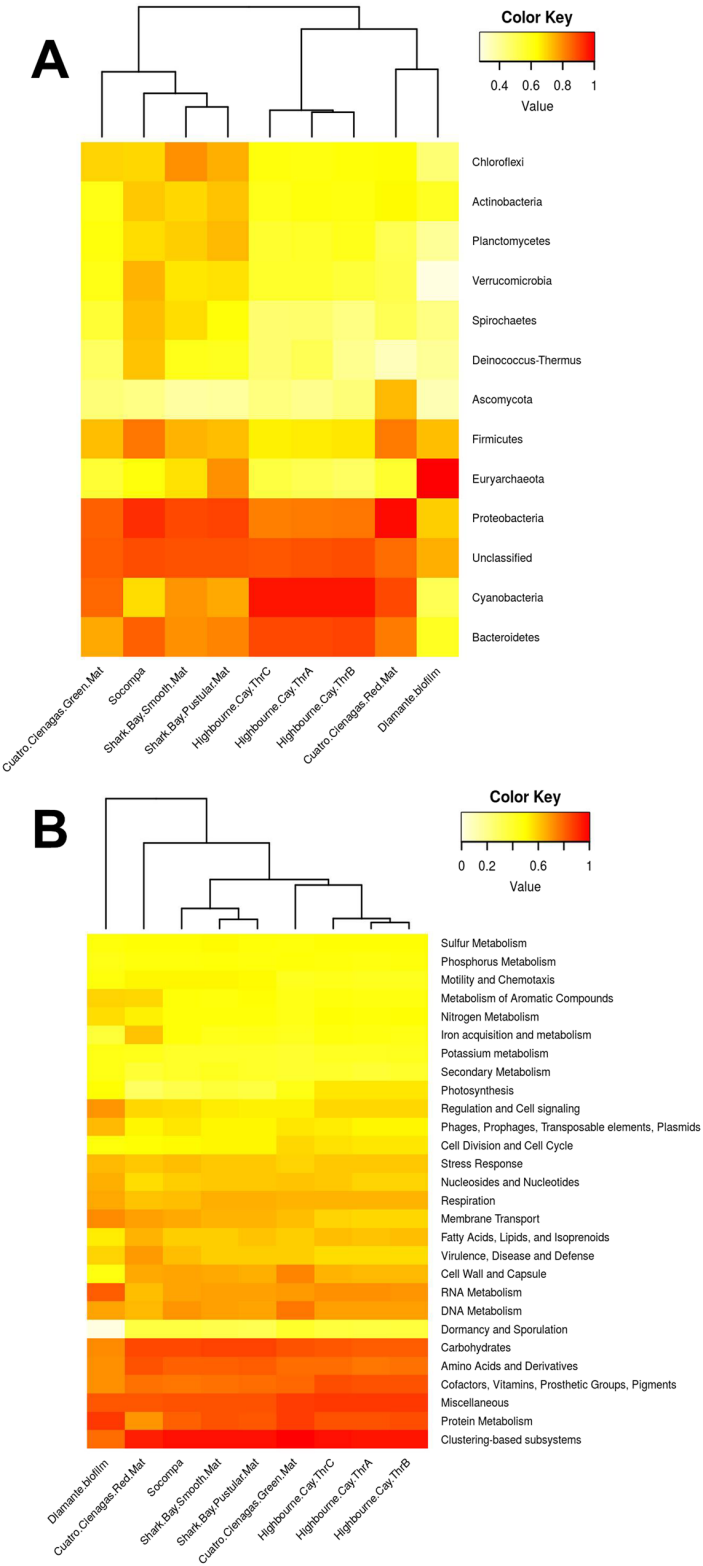
Comparative analysis of several microbial communities at taxonomic phylum level (**A**) and functional level (**B**). Abundance data normalized by MG-RAST is represented with a color gradient from white (less abundant) to red (most abundant). Comparisons were performed with R vegan package and heatmaps were generated with R gplot package.

Shark Bay (Australia)^41^, Highbourne Cay (Bahamas)^46^, and Cuatro Ciénagas (Mexico)^47^, are model systems for studying stromatolite development and function; a direct comparison with Socompa provided insight into the genomic features that make this HAAL environment so unique. Diamante Lake (Argentina)^8^, another HAAL location recently described with even more extreme conditions, was also included in the comparison. By both a taxonomical and a functional analysis on a broad level, Socompa’s metagenome was similar to Shark Bay smooth mats (Fig. 2), while the Red Mat from Cuatro Ciénagas, and Diamante Lake’s archaeal biofilms were more distantly related. This is most likely due to their unusual taxonomic composition dominated by specific taxa (i.e. *Halobacteria* in Diamante and *Pseudomonas* in Cuatro Ciénagas), which is reflected in profiles dominated by functions belonging to such taxa. The Cyanobacteria-dominated Highbourne Cay thrombolites were also different from Socompa’s, in that the former shows a core microbial community structure which induces photosynthetic carbonate precipitation^46^. Still, given the particular combination of extreme environmental conditions, Socompa’s microbial diversity was expected to be unique.

**Figure 3:**
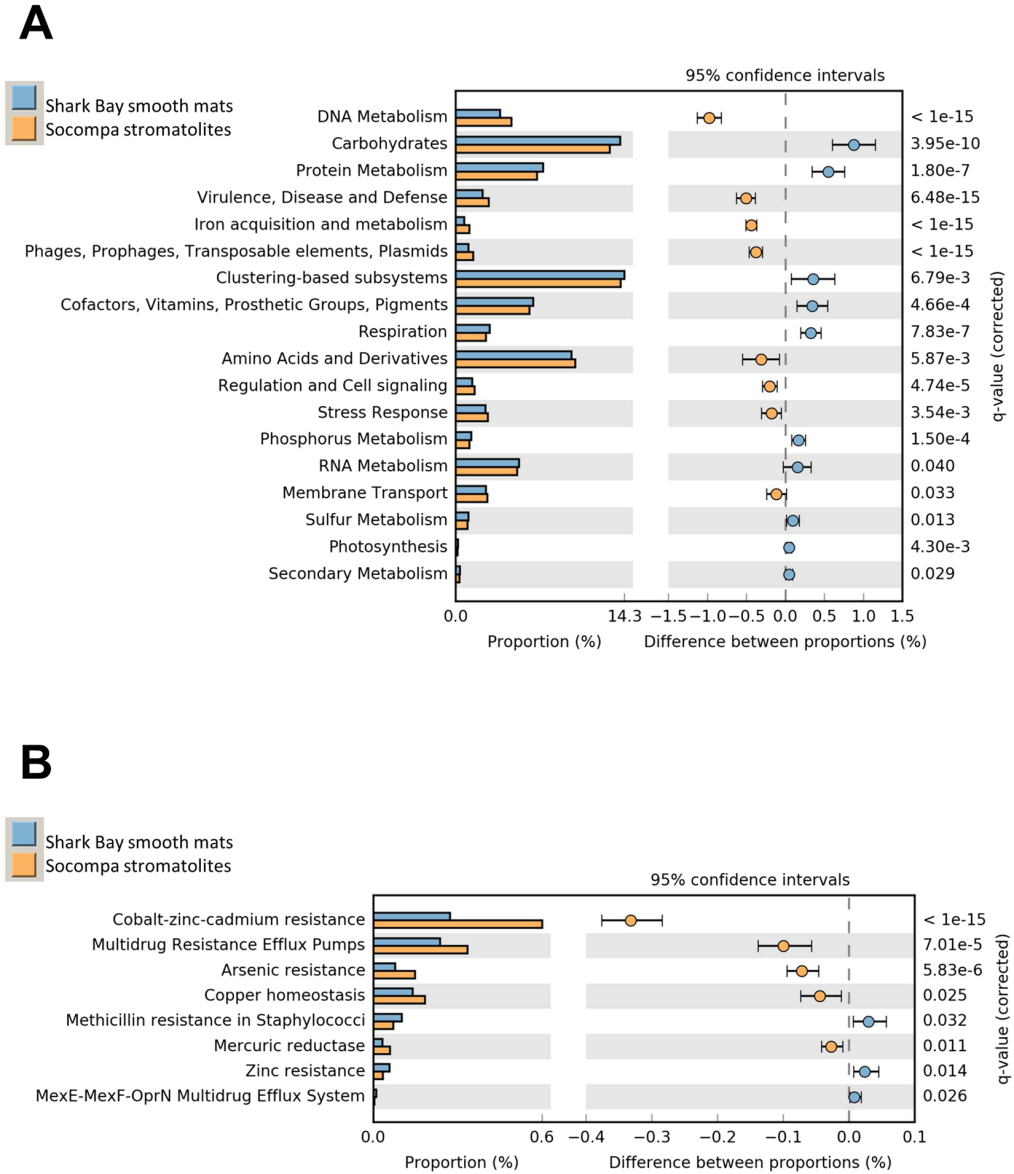
Comparison of Shark Bay smooth mats (blue) and Socompa stromatolites (yellow) by SEED subsystems at level 1 (**A**) and Virulence, defense and disease level 3 (**B**) using STAMP. Features displayed show significant differences.

Since Shark Bay smooth mats was the community most similar to Socompa among the analysed systems, we further compared them to the Socompa stromatolites. Published physicochemical data (Supplementary Table S3) showed that surrounding waters have similar pH (~8) and temperatures (16–32 °C)^19, 48^. Na^+^ and Cl^−^ concentrations are also comparable, although a number of ions such as Ca^2+^ and K^+^ seem to appear in higher concentrations at Socompa. Also, though both sites have a strong UV incidence, it is slightly stronger at Socompa, according to satellite data^3, 49–51^. Further comparison by SEED subsystems using STAMP^52^ showed significant differences at all levels (Fig. 3). Carbon and protein metabolisms were more relevant at Shark Bay, while Socompa was richer in stress-related systems, for example “Virulence, disease and defense”, “Iron acquisition and metabolism”, “Phages, prophages, transposable elements and plasmids”, and “Stress response”. Also, the difference in “DNA metabolism” was related to a great abundance of restriction and modification systems in Socompa (Supplementary Fig. S2), and could be linked to phage resistance or DNA repair. Phages have been reported to be unique in similar environments, such as Cuatro Ciénagas and Highborne Cay^53^, and are reported to influence organomineralization processes^54^, however, their impact on the ecosystem is unknown. DNA recombination systems involved in DNA repair such as RecBCD or RecFOR are more frequent in Socompa, while the UvrABC DNA repair system is more prevalent in Shark Bay. Other UV resistance genes, such as photolyases, show no significant differences. Since both locations are subject to UV radiation stress, the differences might be subtle, but a detailed UV-resistance analysis is beyond the scope of this paper.

The “Stress response” subsystems include “Osmotic stress” genes, most of which are more abundant at Socompa (Supplementary Fig. S3), and could be caused by a number of ions present in the environment, not only NaCl (Supplementary Table S3). Several subsystems involving the biosynthesis of different osmoprotectants are more abundant at Socompa (Supplementary Fig. S3), suggesting more varied strategies for stress resistance. In Shark Bay, the genes involved in betaine biosynthesis from glycine are more abundant, which points at a preference for this compatible solute.

Among the “Virulence disease and defense” group, the genes for “Cobalt-zinc-cadmium resistance and Arsenic resistance” showed a more pronounced difference (Fig. 3B), with over 50% more genes in Socompa, highlighting the effect of the mentioned environmental hazards. Arsenic concentration is very high at Socompa (18.5 mg L^−1^), but there is no data on the contents of other metals^19^. It has been reported that some arsenic genes can be retrieved even from uncontaminated environments^2, 18, 55, 56^. Thus, it is not surprising that they were observed in the Shark Bay metagenomes^41^. However, the extreme conditions at Socompa hinted at the presence of a diverse set of arsenic metabolism genes that will be further analysed in the following sections.

### Arsenic gene diversity

**Figure 4:**
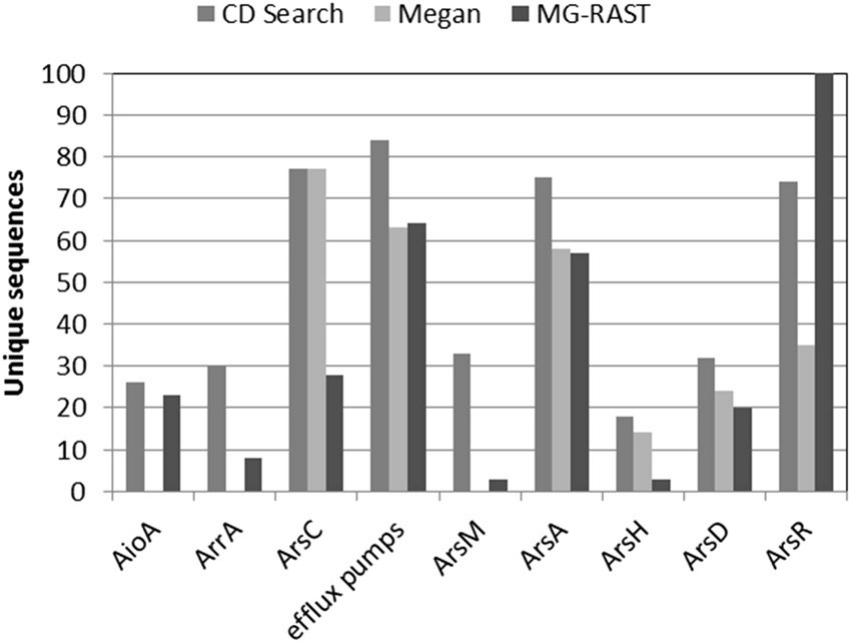
Diversity of arsenic metabolism related proteins, based on NCBI CDD Search, MEGAN, and MG-RAST annotation. Efflux pumps include both Acr3 and ArsB.

Due to magmatic genesis through volcanic activity and the circulation of subterranean water, the Andean region is an environment with some of the highest arsenic contents in the world^33^. Given the high microbial diversity in stromatolites and the isolation of As resistant strains described previously at Socompa and other Andean lakes^8, 19–22^, this study was conducted in order to determine arsenic metabolisms in Socompa stromatolites. To provide an overview of the arsenic biogeochemical cycle in this community, the selected marker genes covering all known arsenic metabolism and resistance pathways included the resistance genes *arsABCDH*, *acr3*, *arsM*, and those related to energetic metabolism *aioA* and *arrA* ^1, 2, 18^. By using several methods, we first compared the number of different proteins that were present, which led to the identification of members of all the selected proteins. The basic arsenic resistance proteins ArsC and the efflux pumps (ArsB and Acr3) were the most diverse, with more than 60 members identified from each class (Fig. 4). Functional annotation of proteins is an acknowledged problem and a primary challenge in molecular biology today^57^; the association between a protein sequence and its function is not often straightforward, and one of the issues is database missannotation^58^. To overcome this problem, three methods were applied in this work, namely MEGAN, MG-RAST and CDD. ArsM, ArrA and AioA are not included in any of the SEED subsystems^59^ and MEGAN was unable to identify them by this approach. The same happened with MG-RAST (data not shown), but this server can also use GenBank annotations which then did produce results. However, counts are based on annotations made with any of the words “arsenic, arsenite, arsenate” and hits with a more general annotation might be missing. This is the case of ArsC, where this method yielded half of the hits, as the thioredoxin dependent family is closely related to the low molecular weight phosphatase family, and many putative ArsC proteins are annotated as phosphatases. To a lesser degree, this might also be the case with ArrA, where some proteins are annotated as “molibdopterin oxidoreductase” or “dehydrogenase”. On the other hand, annotation with NCBI CDD generated most counts. Proteins with an e-value slightly above threshold (Supplementary Table S4) in some cases presented good e-values for other families; therefore, this method might be overestimating the counts. In the case of the PRK11873 ArsM family, where the difference between methods is significant, most of the hits were solid: 27 out of 33 with e-values below 1E-25. As arsenic methyltransferase activity has only recently been reported^60, 61^, it is neither on any SEED subsystem, nor properly annotated on GenBank. Cai *et al*.^2^ used custom databases to annotate arsenic related genes and, using BLAST against its ArsM database, 30 hits were obtained from the Socompa metagenome with e-values below 1E-25, reinforcing the hypothesis that ArsM is widespread at Socompa.

A phylogenetic analysis also underpinned these results, providing further insight. Taxonomic assignments of Socompa proteins were based on sequence homology, but what must be kept in mind is that they might not be assigned to their respective species because of the possible occurrence of horizontal gene transfer^1^. Still, phylogenetic trees were built for several of the marker proteins.

**Figure 5:**
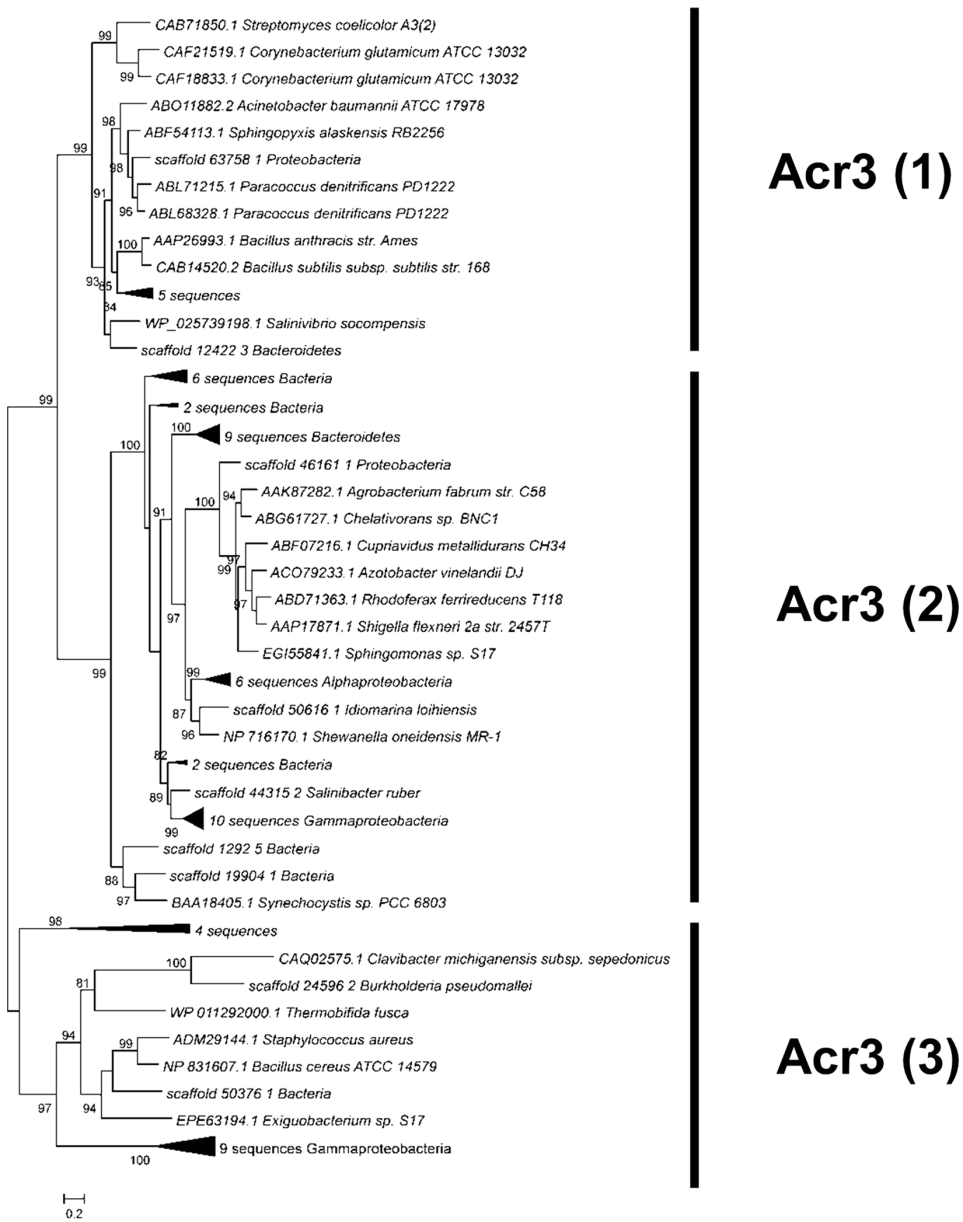
Maximum likelihood tree for Acr3 proteins, including 62 sequences from Socompa and 21 sequences from NCBI nr database. Metagenomic sequences with the same affiliation in the same branch are collapsed. The three groups of the Acr3 family are marked, and all of them include Socompa sequences.
- **ArsC proteins**. The cytoplasmic arsenate reductases (ArsC) are small redox enzymes that reduce arsenate to arsenite by the sequential involvement of three different thiolate nucleophiles that function as a redox cascade. They can be divided into two different classes on the basis of their structures: the thioredoxin-coupled class and the glutaredoxin-linked class^67^. ArsC proteins are fundamental to the reduction of As(V) prior to extrusion. Phylogenetic analyses of short sequences are neither robust nor reliable, so we did not attempt to analyse ArsC sequences. Eight full-length glutaredoxin-linked proteins were identified, most of them belonging to Gammaproteobacteria, such as *Idiomarina* and *Halomonas*. One of the *Halomonas* sequences, Scaffold_18529_3, is a fusion protein with an ArsR DNA-binding regulator domain. A BLAST search against NCBI nr database showed no homologs, which could entail an assembly artifact or a remarkable feature of the environment. Glutaredoxin-linked sequences are also found in strains previously isolated from Socompa, including *Sphingomonas* sp. S17^68^ and *Salinivibrio* sp. S34^21^. The thioredoxin-dependent type is much more diverse in Socompa than the glutaredoxin-linked type, probably because the thioredoxin reducing system is more efficient in arsenate decontamination^69^. A similar observation was made by Escudero *et al*.^18^, who found glutathione-dependent ArsC only in low arsenic content sites. One of the most arsenic resistant isolates in Socompa, *Exiguobacterium* sp. S17, bears the ArsC type^22, 70^.
- **AioA and ArrA** are proteins that belong to the DMSO reductase family or complex iron–sulfur molybdoenzyme (CISM) family^71^. They catalyze arsenic oxidation (AioAB) or reduction (ArrAB) in membrane-associated processes that couple with energy generation^72^, and are thus called arsenic respiratory oxidases and reductases. These enzymes are frequently present in environments where arsenic is rife. In Archaea, they were reported for the first time at Diamante –another HAAL located in the caldera of Galán Volcano– with an As content in water of 119 mg L^−1^ ^8^. The respiratory oxidases and reductases are large proteins, with usually at least 800 residues, so only a limited number was available for phylogenetic trees, as complete genes could only be found in the larger contigs. Even though 26 AioA hits were found in Socompa, most were incomplete proteins and a phylogenetic analysis was not carried out. Scaffold_126_14 was classified as Thermaceae family, with only 63% identity to its BLAST best hit in the NCBI nr database. Scaffold_4942_3 was classified as *Marinobacter* genus, with 89% identity to its BLAST best hit. For the ArrA proteins, 9 full-length proteins were analysed out of 30 hits, whereby a phylogenetic tree was generated (Fig. 6). Two groups can be identified in the tree, ArrA and ArxA proteins. Scaffold_4302_4 diverged in that it was found similar to another group of the DMSO reductase family, the PHLH –putative hydrogenase like homologs–group (data not shown), and was thus used as root. The ArxA group includes proteins from a recently described clade that is closely related to ArrA proteins^73^. Rather than reducing arsenate, ArxA proteins oxidized arsenite. Scaffold_2655_6 was included in this group and affiliated to *Halomonas*. The ArrA proteins included seven members from Socompa. Two proteins were assigned to *Alkaliphilus* and grouped with Firmicutes and Archaea. The remaining ones were loosely classified: two were distantly related to a Gammaproteobacteria group, and the last three were included in a diverse group, with sequences from Betaproteobacteria, Deltaproteobacteria, and Firmicutes. The number and diversity of the identified proteins suggest that many microorganisms in this environment are capable of using arsenic as an energy source. This mechanism could be widespread in high altitude Andean arsenic systems, as shown in findings at Diamante Lake^8^.

- **The efflux pumps ArsB and Acr3**. Only one protein in the entire metagenome was classified as an ArsB efflux pump, affiliated to the Bacillales order. Acr3 was much more diverse and a phylogenetic tree was built with 62 sequences from Socompa and 21 sequences from databases (Fig. 5). The Acr3 proteins are part of the bile/arsenite/riboflavin transporter (BART) superfamily^62^. All members of this superfamily bear a basic five-transmembrane segment region; the Acr3 family has two such regions. Acr3-like proteins have been classified into three subgroups, which are independent of taxonomic affiliation. Groups Acr3(1) and Acr3(2) have functionally characterized members^63–66^. Group Acr3(3), whose members are less closely related, is less compact and basically includes all proteins that do not fit into the other groups. No member from the latter group has been functionally characterized, although *Exiguobacterium* sp. S17, a highly arsenic-resistant strain from Socompa Lake, has been shown to encode Acr3(3). It was hypothesized that, since all known *Exiguobacterium* strains have a conserved *arsB* gene, and S17 is the only one bearing both genes, the presence of an additional pump might explain its resistance profile^22^. Forty Socompa proteins could be assigned to the Acr3(2) group, which comprises mostly proteins from Proteobacteria, the most abundant phylum in the metagenome. Fifteen proteins belonged to Acr3(3) and seven to Acr3(1). Scaffold_12422_3 from Acr3(1) grouped closely with WP_025739198.1 from *Salinivibrio socompensis*, isolated from the stromatolites^21^.

**Figure 6:**
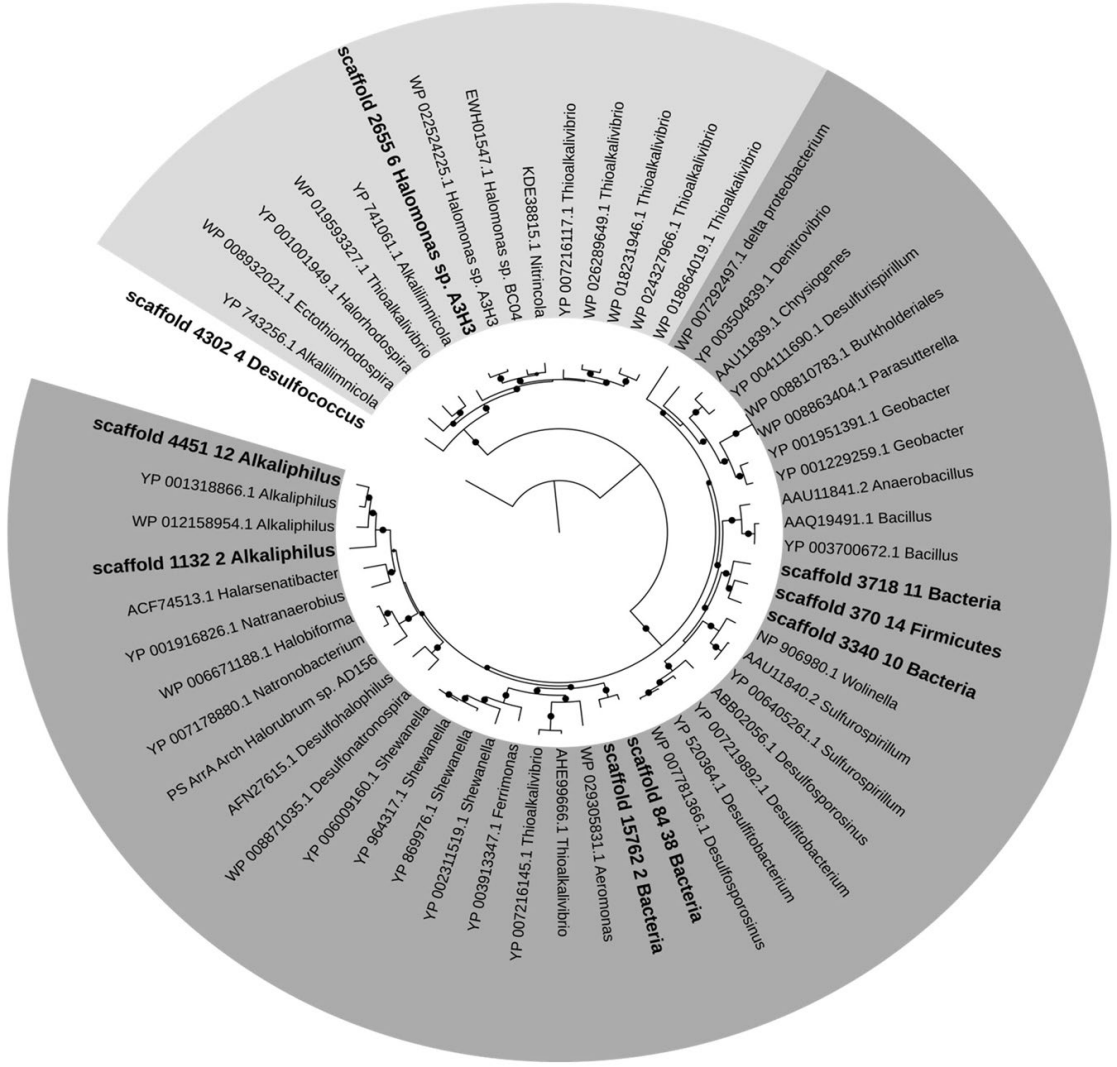
ArrA subfamily maximum likelihood tree. The arsenite oxidases clade ArxA is shown in light grey and the arsenate reductases in dark grey. The tree is rooted with scaffold_4302_4, which belongs to another DMSO reductases subfamily. Socompa sequences are in bold. Nodes with bootstraps above 0.7 are marked with a filled circle.
- **ArsM**. This protein belongs to the UbiE/Coq5 C-methyltransferase family and it is characterized by a conserved cysteine residue in the active site. It is an As(III) S-adenosylmethionine methyltransferase responsible for As(III) methylation^72^. Although these proteins should have a common function, the sequences are quite different, and the group includes members of all three kingdoms. The ArsM tree included 19 Socompa sequences and 33 database sequences, as shown in Supplementary Fig. S4 where the proteins whose function has been experimentally confirmed have labels in bold. Many of the sequences belonged to Bacteroidetes and were closely related, clustering together in the same branch. However, Socompa sequences were found throughout the tree, suggesting ample diversity.

**Figure 7:**
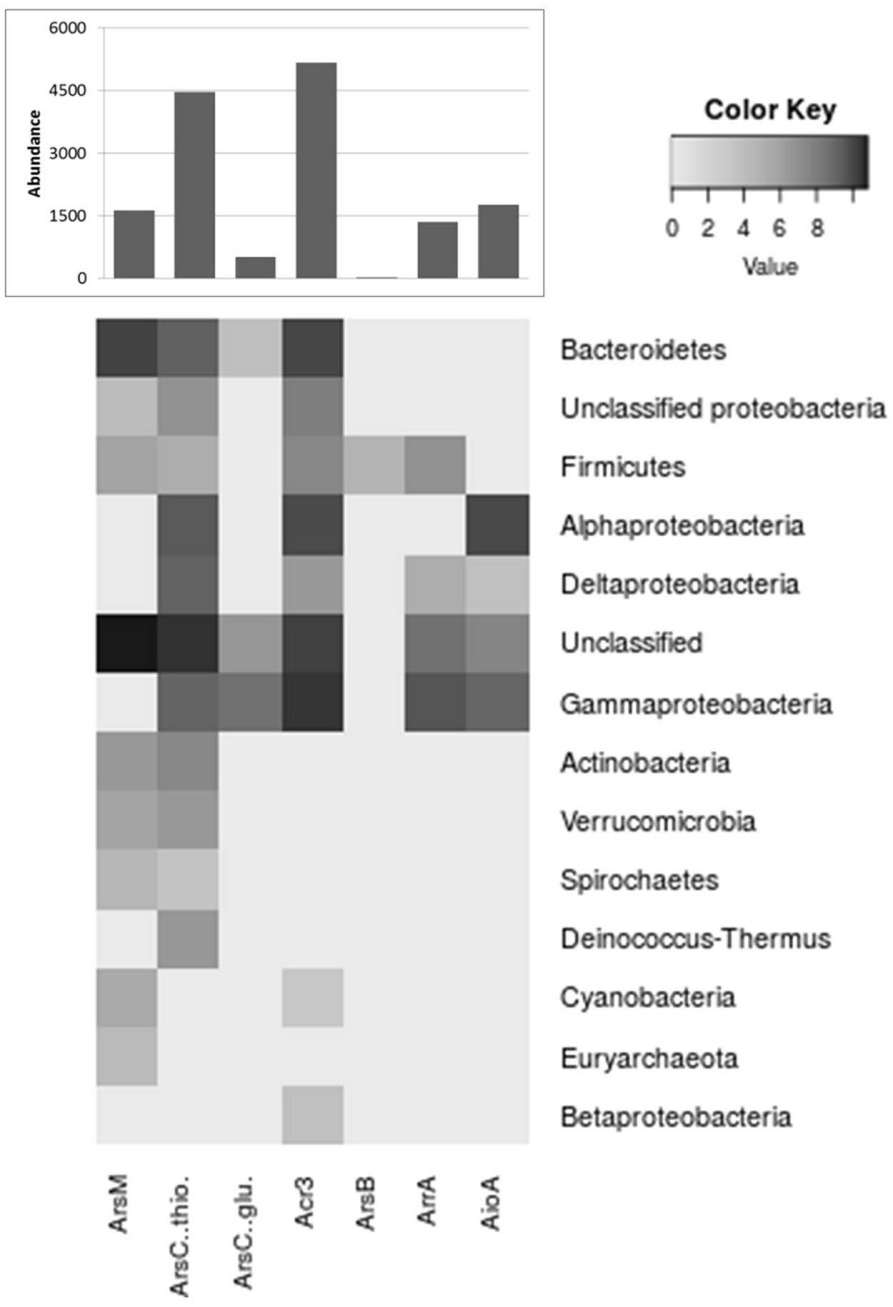
Arsenic genes abundances and affiliation. Counts were log-transformed (log_2_(x + 1)) and drawn as a heatmap.

The phylogenetic analysis only included genes encoding complete, full-length proteins. For a more comprehensive view, we also analysed the abundance of all identified proteins. As the genes were taxonomically classified into different levels, we analysed them at the phylum level. The abundance pattern (bars in the upper part of Fig. 7) is similar to the diversity pattern shown in Fig. 4. The Acr3 efflux pump and the ArsC thioredoxin-dependent reductase-encoding genes are the most abundant ones, encompassing more than 60% of the sequences. Acr3 from Gammaproteobacteria was the most profuse, followed by Bacteroidetes, Alphaproteobacteria and the “unclassified” group. Thioredoxin-dependent ArsC showed a similar trend, although sequences were distributed among other phyla as well. Gluthathion-dependent ArsC sequences belonged mostly to Gammaproteobacteria, probably from *Idiomarina* and *Halomonas*. The only representative sequence of ArsB efflux pump belonged to Firmicutes.

The energy generation associated genes ArrA and AioA were more abundant among Proteobacteria, mostly Gammaproteobacteria, but ArrA was abundant in Firmicutes and Deltaproteobacteria, which are likely to thrive in an anoxygenic environment. AioA was mostly affiliated to Alphaproteobacteria, where, as a chemical energy source, As[III] might help anoxygenic photosynthesizers for primary production. Also, the Gammaproteobacteria *Marinobacter* is one of the most frequent genera within this metagenome, perhaps associated to an efficient energy generating system based on *aio* genes. Finally, ArsM showed an interesting distribution. Most sequences were unclassified, followed by Bacteroidetes members, and no representatives of Proteobacteria, although these might appear among the unclassified group. What is more, ArsM was found in most phyla, even where the most abundant Acr3, such as Actinobacteria, Verrucomicrobia, Spirochaetes, Euryarchaeotes, was not found. These are among the least abundant, and it is either this or the failure to classify it properly that might cause Acr3 absence. The *arsM* gene is smaller and might have been identified in the shorter contigs from these rare phyla. Another possibility is that ArsM might represent the main alternative arsenic resistance mechanism, producing volatile or less toxic compounds such as monomethylarsonate, dimethylarsonate, trimethylarsenic oxide and trimethylarsine, though the more toxic intermediates –mono and di-methyl arsine– might also be produced^74^. Finally, it is also plausible that part of the community simply relies on the detoxification abilities of other members. This has been concluded in another study using a metagenomic approach in a simple community from an acid mine drainage^75^, but it would be difficult to assess such cooperation in a much more diverse environment as in the case of Socompa stromatolites.

### Binning

Automatic binning was attempted by means of Metawatt 3.2^76^. Four high quality, four medium quality, and 21 partial bins were generated. These bins were further polished manually by eliminating contigs assigned to different phyla within a bin, or with widely different sequence coverage. Three bins were selected for further analysis: H, J, and Y (Supplementary Fig. S5). These bins could represent dominant organisms found in stromatolites, with abundance levels below 5–10%, according to a 97% similarity OTU classification. However, more than one closely related organism might be represented in the same bin. For further analysis, the bins were uploaded to the RAST server^59^. Partial SSU RNA genes found in the bins were compared to the NCBI 16S database by BLAST, which yielded a different result from the whole genome closest neighbour analysis by RAST (Table 1).

**Table 1:** Bin characteristics and similarities to known organisms.

	Bin	Closest neighbors (RAST)		partial 16S rRNA gene BLAST best hit
	**Y**	*Chromohalobacter salexigens* (Gammaproteobacteria)	*Halomonas elongata* (Gammaproteobacteria)	*Desulfotignum balticum* (Deltaproteobacteria)
Size (Mb)	4.8	3.7	4.06	5.12
%GC	52.7	63.9	63.5	51.2
	**H**	*Gramella forsetii* (Bacteroidetes)	*Croceibacter atlanticus* (Bacteroidetes)	*Zunongwangia profunda* (Bacteroidetes)
Size (Mb)	4.76	3.8	2.95	5.13
%GC	34.9	36.6	33.9	36.2
	**J**	*Desulfococcus oleovorans* (Deltaproteobacteria)	*Desulfatibacillum alkenivorans* (Deltaproteobacteria)	*Desulfococcus multivorans* (Deltaproteobacteria)
Size (Mb)	4.6	3.94	6.52	4.4
%GC	52.7	56.2	54.5	56.8

Bin H was classified as a high quality bin that bears relation to *Gramella forsetii*, with a larger genome size, but similar GC content, and it is likely a member of the Flavobacteriaceae family, Bacteroidetes phylum. Even though the ArsM protein was abundant within Bacteroidetes (Fig. 7), it was absent from this bin. Although it would be difficult to assign a specific function to this organism, we can speculate that it might be involved in the degradation of high molecular weight compounds that probably derive from EPS, as several polysaccharide utilization loci (PULs)^77^ with unknown specificity are observed. Such function might be involved in stromatolite formation, since EPS degradation liberates Ca^2+^ and HCO_3_^−^ ions, which contribute to carbonate precipitation^78^, but again, at this point the assumption remains speculative. It should be mentioned that *Gramella forsetii*, among other Bacteroidetes, plays a role in the degradation of algal glycans in marine ecosystems^79^.

Bin J was related to Deltaproteobacteria of the genus *Desulfococcus*, which in all likelihood contribute to sulfate reduction; as a matter of fact, dissimilatory sulfite reductase and related genes^80^ are present in this bin. This process is important as it is one of the main boosters behind the formation of hypersaline mats^78^, and in Socompa it could be one of the processes that influences the formation of the stromatolite structure^19^. A recent differential metagenomic study identified sulfate reduction, arsenate reduction and fermentation as major functions in a microbial community inhabiting arsenic-contaminated marine sediments^81^. Microbial arsenic precipitation has been associated to sulfate reduction in sites with high arsenic content^16, 18^. Since bin J encodes genes involved in sulfate reduction, but does not bear an *arrA* gene, it might not be involved in precipitation. Mineralogy data from Socompa stromatolites do not show arsenic minerals^28^ indicating that arsenic does not precipitate in these stromatolites at all. However, a small subset of incomplete *arrA* sequences are affiliated to Deltaproteobacteria and Firmicutes (Fig. 7), suggesting that some members of the microbial community might be able to precipitate arsenic under controlled conditions, like *Desulfosporosinus auripigmentum* ^82, 83^, *Desulfovibrio* strain Ben-RA^84^, or enrichment cultures from high arsenic content sites in northern Chile^16^. In addition to sulphate reduction, bin J includes nitrogenase genes, probably involved in nitrogen fixation, which would demonstrate another relevant role played by these members of the community. Although the biological function of nitrogenases is the conversion of nitrogen gas to ammonia, these enzymes also generate hydrogen gas as an obligatory aspect of their catalytic mechanism^85^, which –upon reconversion into protons and electrons– could serve as an electron source either for sulfate reduction or other metabolic processes.

Bin Y was assigned to Gammaproteobacteria. The closest match in RAST was *Chromohalobacter salexigens*. However, the GC content is markedly different than that observed for the three known *Chromohalobacter* species, adding to the fact that the genome is larger in size. GC content values are more consistent with the second hit, *Halomonas elongata*. Having been sequenced, many members of the *Halomonas* genus have shown a genome GC content that varies between 52 to 68%, and whose size ranges from 2.8 to 5.9 Mb. About 40% of the bin’s protein hits matched the *Halomonas* genus, and 10% matched *Chromohalobacter*. In spite of this, a full 16S rRNA gene found in this bin matched *Desulfotignum balticum* (Deltaproteobacteria) with 96% identity. Thus, even though the coverage of the contigs is higher in this bin than in the other two (bins J and H), it might include contigs from more than one species.

The bins were further analysed to assess arsenic resistance mechanisms (Supplementary Fig. S6). All of them presented a copy of the *acr3* extrusion pump and an *arsC* reductase, thus covering the basic arsenic resistance mechanism. Bins H and Y comprise additional genes, including regulators and *arsH*, organized in operons. Bin Y presented two copies of *acr3* that are also present in reference *Halomonas* genomes, but the existence of two copies might indicate increased resistance required to survive in Socompa’s high-arsenic environment. The *arsM* system is absent, being perhaps the system of choice in less abundant organisms. Neither *arrA* nor *aioA* genes were found, even though the *arxA* scaffold_2655_6 was affiliated to *Halomonas*. However, the *arxA* gene in *Halomonas* sp. A3H3 is present in a plasmid^86^, and this might have been missed by the binning procedure used. Also, other incomplete bins associated with *Halomonas* were observed during binning, which suggests the presence of several species in different abundances. Even though we have not considered arsenic uptake in this work, a periplasmic phosphate binding protein (PBP) with high selectivity for phosphate over arsenate has been proposed as a resistance mechanism in the *Halomonas* sp. GFAJ-1 isolated from the arsenic-rich Mono Lake^87^. A protein with 89.4% identity to GFAJ-1 PBP assigned to *Halomonas* is present in Socompa’s metagenome, and another protein with 79.5% identity is present in bin Y. Thus, phosphate selectivity would be an additional adaptation mechanism present in the *Halomonas* population in Socompa. Phosphorous concentration at Socompa is high, eight times higher than arsenic in the stromatolites, and about the same ratio is found in water^28^. However, it cannot be stated whether the different concentrations of elements ameliorates arsenic’s toxic effects.

## Conclusion

**Figure 8:**
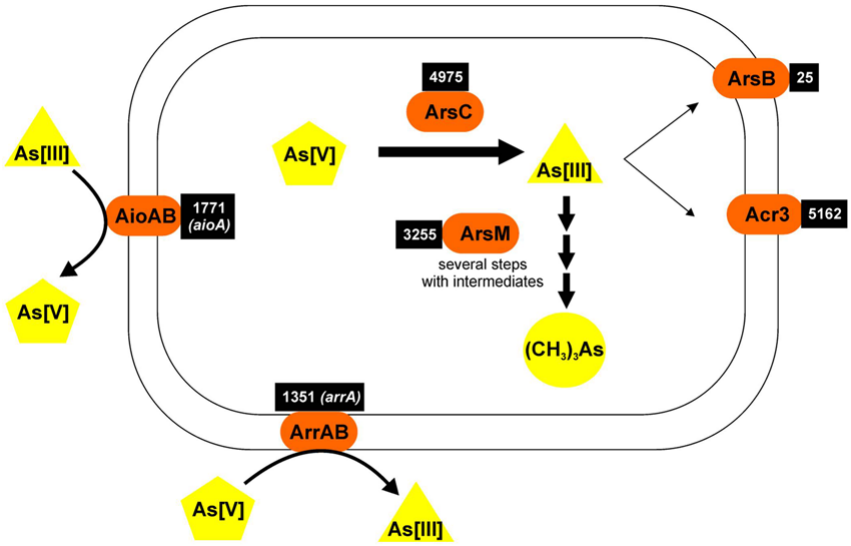
Summary of the arsenic metabolism in Socompa stromatolites. Abundances of the genes are shown in black boxes. *arxA* sequences are counted among the *arrA*, although they perform As[III] oxidation.

A high arsenic concentration is only one of the extreme conditions acting as selective pressure on Socompa stromatolites. Stratified microbial communities known as stromatolites thrive in a poly-extreme environment, in spite of which they are surprisingly diverse, as revealed by the metagenomic analysis. While they share common determinants with other mat and stromatolite communities, they also show unique characteristics, related to environmental stress. Their arsenic resistance mechanisms are based mainly on reduction and extrusion of As(V) by diverse Acr3 efflux pumps, associated to ArsC (thioredoxin type) reductases, which is also observed in Socompa isolated strains (Supplementary Fig. S6). However, a complete set of arsenic metabolism genes is present, including ArsM methyltransferases from the most varied phyla, as well as respiratory AioA oxidases, ArrA reductases, and even the novel ArxA oxidases, all summarized in Fig. 8. Thus, the community is capable of a complete arsenic cycle, even using it as an energy source. The binning of the assembled contigs illustrates that arsenic resistance is important for growth since the most abundant organisms –represented by the selected bins– carry resistance genes. A few notorious genes from the bins have been highlighted, including genes for sulfate reduction, polysaccharide utilization, and selective phosphate pumps, making clear allusion to their prominent roles in the complex stromatolite community.

This metagenome renders powerful as a tool to guide further studies on the identification of unique traits of the microbial community and on the functional characterization of these stromatolites which otherwise represent a modern analogue to their Precambrian counterparts. A detailed layer-by-layer study by metagenomics and experimental methodologies will help define the spatial distribution of the different metabolic processes within the stromatolite.

## Experimental procedures

### Sampling

Stromatolites can be found along the southern shore of Socompa Lake, at 24°32.168′S, 68°12.165′W. The site was exposed to air at the moment of sampling (February 2011), but it is submersed under 0.5–1 m of water from about May to December. These stromatolites are rounded domal structures that present a clear stratification that appear in vertical sections (Fig. 1A and B). Stromatolite samples were collected in sterile plastic bags and were fragmented into 2.5 cm-diameter cylinders that measured 5 cm deep from the top of the stromatolite. They were frozen in liquid nitrogen, and processed within a week.

Permission for sample collection was granted by the Ministerio de Ambiente y Producción Sustentable, Salta, Argentina (number 000388; 17–09–2010).

### DNA extraction and sequencing

Frozen stromatolite samples were lyophilized and homogenized into a powder that was processed as previously reported^19^. Three replicate extractions were pooled and sequenced. DNA was sheared by sonication into fragment sizes of around 240 bp, it was cleansed (Zymo Research), and universal sequencing adaptors were ligated. After library quantification at a Qubit (Invitrogen), a 10 nM stock solution of the amplified library was created. Sequencing-by-synthesis was performed on an Illumina Genome Analyzer (GAIIx). Two test lanes with 36 bp single reads and one lane with 120 bp paired-end reads were run. Raw data is available from NCBI’s SRA under BioProject PRJNA317551.

### Assembly, annotation, and binning

Raw reads from all three lanes were quality-filtered and trimmed with Sickle^88^, using default parameters (>Q_20_, read length >20 bp). Filtered reads (71,359,349 singles and 79,477,482 paired-end totalizing 12,319,219,512 bp) were first analysed with Nonpareil^35^ to estimate coverage and diversity. Assembly of the filtered reads was performed with IDBA_UD^36^, including all three lanes. Assembled contigs >1000 bp were annotated with Prodigal^89^, and each ORF was compared to the NCBI nr database using Diamond^90^. Results were analysed with MEGAN5^91^. The NCBI CDD database^92^ was also used for classification of proteins in families. Arsenic-related proteins were selected for further analysis. Supplementary Table S4 shows the CD family, the protein name, and the threshold used for selection. Thresholds were adjusted by BLAST analysis of a few selected proteins above and below threshold. Contigs were uploaded for online analysis with the MG-RAST server v3.6^37^, and data is available by accession number 4626208.3. Finally, contigs were subjected to unsupervised binning with MetaWatt 3.2^76^. Quality of the bins was assessed by the software based on conserved single copy genes (cscg) content: high quality bins cscg >90%, cscg duplicated <15%; medium quality bins cscg >80%, cscg duplicated <25%; partial bins cscg >50%. Selected bins were further cleaned manually and uploaded to RAST^59^ for annotation.

### Metagenomic data comparisons

Metagenomic abundance data was obtained from and normalized with the MG-RAST server using API^93^. Metagenomic datasets used for comparison (with their associated MG-RAST IDs) included Green Mat (4441347.3) and Red Mat (4442467.3) from Cuatro Cienegas, México^47^, Diamante Lake biofilms from Cerro Galán, Catamarca, Argentina (4513627.3)^8^, Shark Bay Smooth (4532715.3) and Pustular (4532716.3) mats, and Highbourne Cay thrombolites (ThrA to C assemblies - 4532758.3 to 4532760.3)^41^. Taxonomic data annotated by the M5NR database was normalized at phylum level, and functional data classified by Subsystems was normalized at Level 1. Normalized data was analysed with R version 3.3.1^94^. The R vegan package^95^ was used to calculate Bray Curtis distances among samples. These distances were used to group samples using complete clustering. Heatmaps were generated with R gplots^96^ and Rcolorbrewer^97^ packages.

Statistical analysis was completed using Statistical Analysis of Metagenomic Profiles (STAMP) version 2.1.0 ^52^. The STAMP Fisher’s exact test was carried out in two samples using a two-sided test, the Newcombe-Wilson method for calculating CIs, and multiple test correction using Storey’s FDR.

### Phylogenetic analysis

Arsenic genes were identified from the annotations, and the corresponding proteins were filtered based on CD Search results. Only proteins with a complete conserved domain were used for phylogenetic analysis, thus, not all of the hits were included in the phylogenetic trees. Selected proteins were aligned using Muscle v3.8.31^98^. From the alignments, maximum likelihood trees were generated with FastTree version 2.1.7 SSE3^99^. Tree figures were drawn using MEGA 6^100^, and iToL 3.0^101^.

## Electronic supplementary material

Supplementary information
