# Supplementary material for: Arsenic metabolism in high altitude modern stromatolites revealed by metagenomic analysis
Source: Sci Rep. 2017 Apr 21;7:1024. doi: 10.1038/s41598-017-00896-0 (PMC5430908; doi:10.1038/s41598-017-00896-0)
Supplement: Supplementary file 1 — Supplementary information [file 41598_2017_896_MOESM1_ESM.pdf]

Supplementary information

Arsenic metabolism in high altitude modern stromatolites revealed by metagenomic analysis

Daniel Kurth[1], Ariel Amadio[2], Omar F. Ordoñez[1], Virginia H. Albarracín[1,3], Wolfgang Gärtner[4], María E. Farías[1*]

[1]Planta Piloto de Procesos Industriales y Microbiológicos (PROIMI), CCT-Tucumán, CONICET, San Miguel de Tucumán, Argentina.
[2]E.E.A. Rafaela, Instituto Nacional de Tecnología Agropecuaria (INTA), Rafaela, CCT Santa Fe, CONICET, Argentina.
[3]Facultad de Ciencias Naturales e Instituto Miguel Lillo, Universidad Nacional de Tucumán, San Miguel de Tucumán, Argentina.
[4]Max-Planck Institute for Chemical Energy Conversion, Mülheim an der Ruhr, Germany.

Supplementary Table S1. A. Assembly statistics for Socompa metagenome. B. Size distribution of contigs.

A

	**All**	**Filtered***
**Contig number**	391,949	79,915
**Total bases**	374,092,121	219,061,845
**Average**	954	2,741
**Longest**	239,159	239,159
**N50**	1,359	3,413
**L50**	52,272	13,984
**N90**	401	1,230
**L90**	276,717	60,096

*Filtered contigs are >1kbp

B

**Contig size**	**Contig number**
> 5 kbp	7808
> 10 kbp	2419
> 50 kbp	92
> 100 kbp	18

Supplementary Table S2. Abundance of selected COGs for carbon fixation, sulfate reduction and nitrogen cycle in Socompa metagenome.

Process	COG ID	function	COG abundance
Carbon fixation	COG1850	RuBisCO large chain, key enzyme for the CBB [EC:4.1.1.39]	230
	COG2301	ATP–citrate lyase, enzyme representing the Arnon–Buchanan cycle (rTCA) [EC:2.3.3.8]	1655
	COG2368	4-hydroxybutyryl dehydratase, key enzyme for the HP/HB and DC/HB cycles [EC:4.2.1.120]	76
	COG1614	acetyl-CoA synthase [EC:2.3.1.169] (acsB)	235
Sulfate reduction	COG2221	Dissimilatory sulfite reductase (desulfoviridin), alpha and beta subunits	117
	COG2046	ATP sulfurylase (sulfate adenylyltransferase)	1512
Nitrogen cycle	COG1251	NAD(P)H-nitrite reductase	430
	COG5013	Nitrate reductase alpha subunit	1307
	COG1140	Nitrate reductase beta subunit	738
	COG3005	Nitrate/TMAO reductases membrane-bound tetraheme cytochrome c subunit	316
	COG3303	Formate-dependent nitrite reductase periplasmic cytochrome c552 subunit	240
	COG4263	Nitrous oxide reductase	370
	COG3256	Nitric oxide reductase large subunit	1574
	COG1348	Nitrogenase subunit NifH (ATPase)	1066

Supplementary Table S3. Physicochemical data for water surrounding stromatolites and mats. Socompa data taken from reference 19. Shark Bay data taken from reference 48.

	Socompa	Shark Bay	
	stromatolites	Nilemah pustular	Nilemah smooth
**Temperature (ºC)**	20-24	16.4-32	17.3-30.8
**pH**	8.6	7.8-8.5	7.5-8.4
**Salinity**	-	62.3–65.3	61.2–65.0
**Conductivity (mS cm^{-1})**	115	-	-
**Sodium (mg L^{-1})**	37113	35900	17,600
**Potassium (mg L^{-1})**	5298	1140	672
**Magnesium (mg L^{-1})**	4090	3680	2050
**Calcium (mg L^{-1})**	2383	1080	577
**Chloride (mg L^{-1})**	52234	64000	32100
**Sulfate (mg L^{-1})**	31847	-	-
**Total Sulfur (mg L^{-1})**	-	3130	1660

Supplemetary Table S4. Families and thresholds used for NCBI CDD assignation.

CD family ID	Gene name	function	Threshold
COG0798	Acr3	efflux pump	1.00E-10
cd01118	ArsB	efflux pump	1.00E-05
cd02757	ArrA	arsenate reductase respiratory, includes Arx oxidase	1.00E-05
cd02756	AioA	arsenite oxidase respiratory	1.00E-05
cd03034	ArsC-glutation dependent	arsenate reductase	1.00E-10
TIGR02691	ArsC-thioredoxin dependent	arsenate reductase	1.00E-10
PRK11873	ArsM	arsenic methylase	1.00E-10
cd02035	ArsA	ATPase	1.00E-19
TIGR02690	ArsH	Unknown – proposed NADPH-dependent FMN reductase	1.00E-10
pfam06953	ArsD	Arsenical resistance operon trans-acting repressor	
cd00090	ArsR	Arsenical resistance operon repressor	1.00E-05

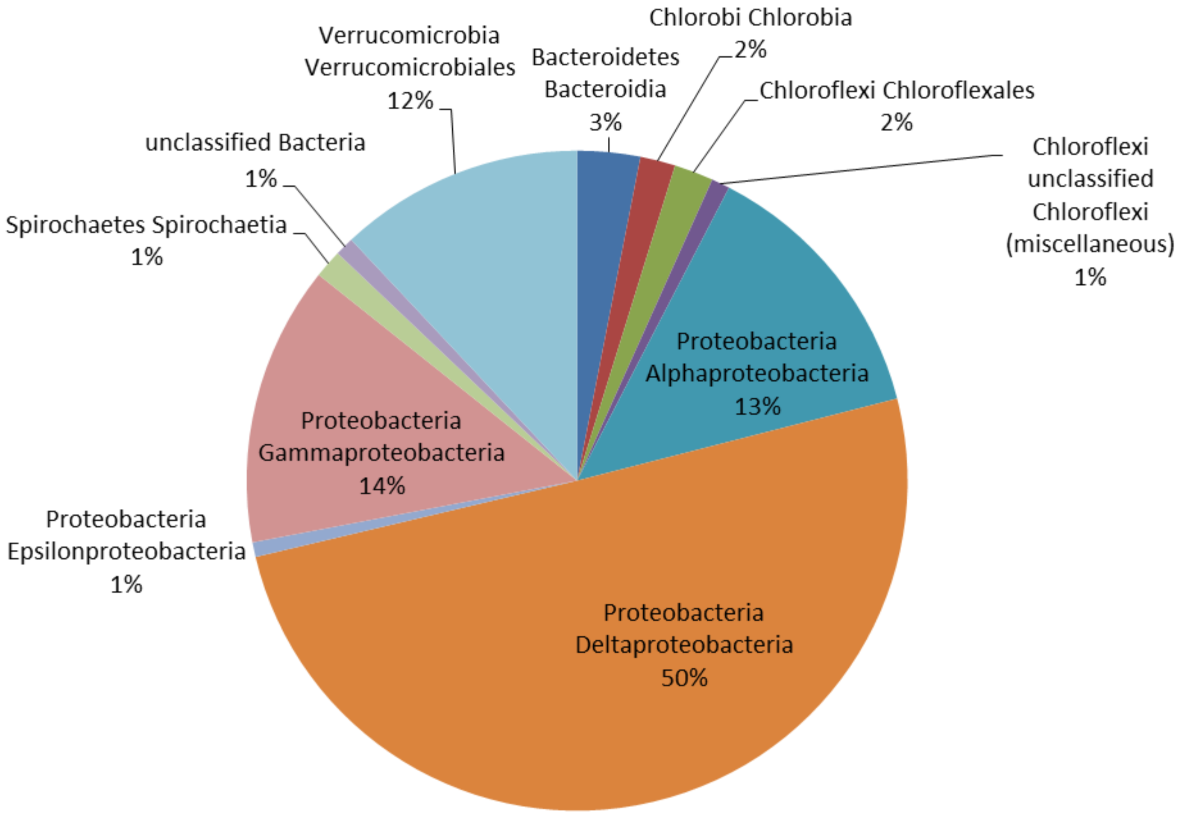

Supplementary Figure S1. Taxonomic affiliation (phylum and class) of nitrogenase sequences, based on BLAST best hits against NCBI nr database.

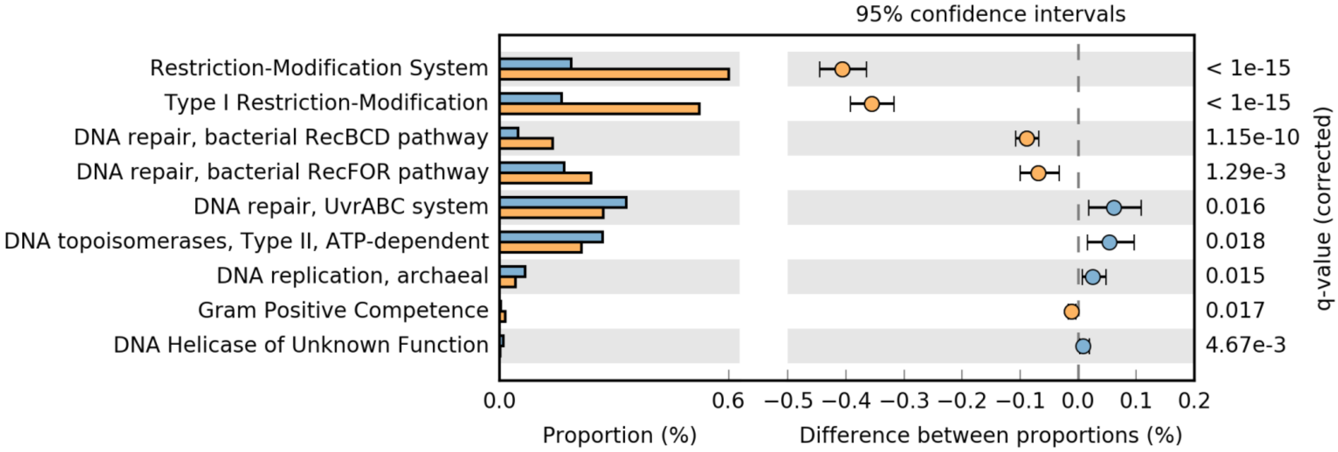

Supplementary Figure S2. Comparison of Shark Bay smooth mats and Socompa DNA metabolism classified by SEED subsystems at DNA metabolism level 3 using STAMP. Features displayed show significant differences.

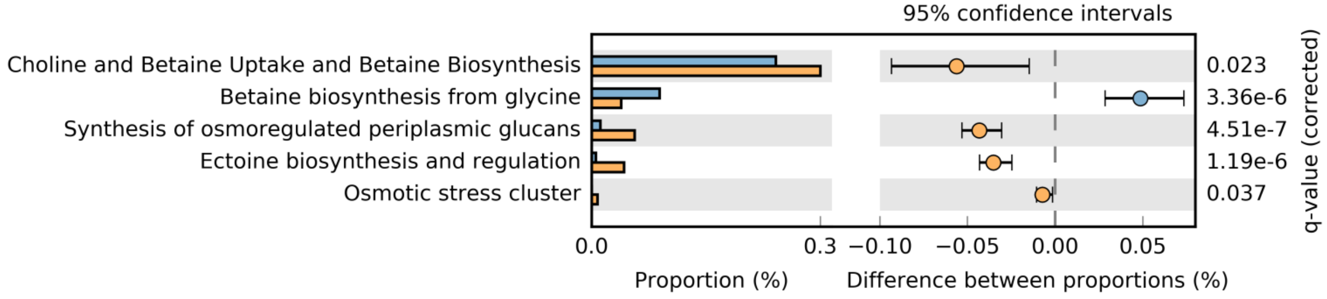

Supplementary Figure S3. Comparison of Shark Bay smooth mats and Socompa DNA metabolism classified by SEED subsystems at Stress reponse, Osmotic Stress level using STAMP. Features displayed show significant differences.

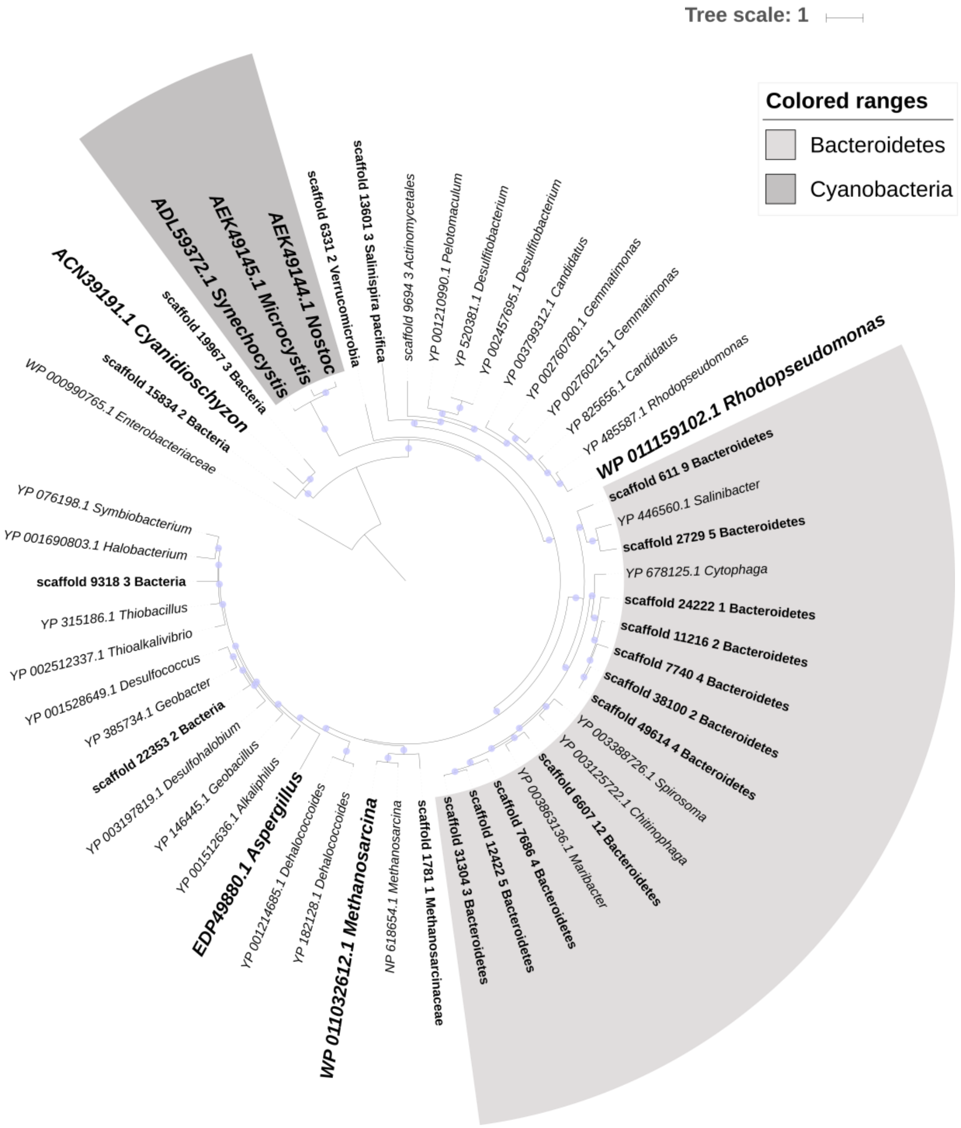

Supplementary Figure S4. ArsM arsenite methyltransferases tree. The tree is rooted with another type of methyltransferases. Nodes with bootstraps above 0.7 are marked with a circle. Groups phylogenetically homogeneous are shaded. Proteins whose activity has been experimentally confirmed have bigger font labels. Socompa proteins are named scaffold_xxxxx and have bolded labels.

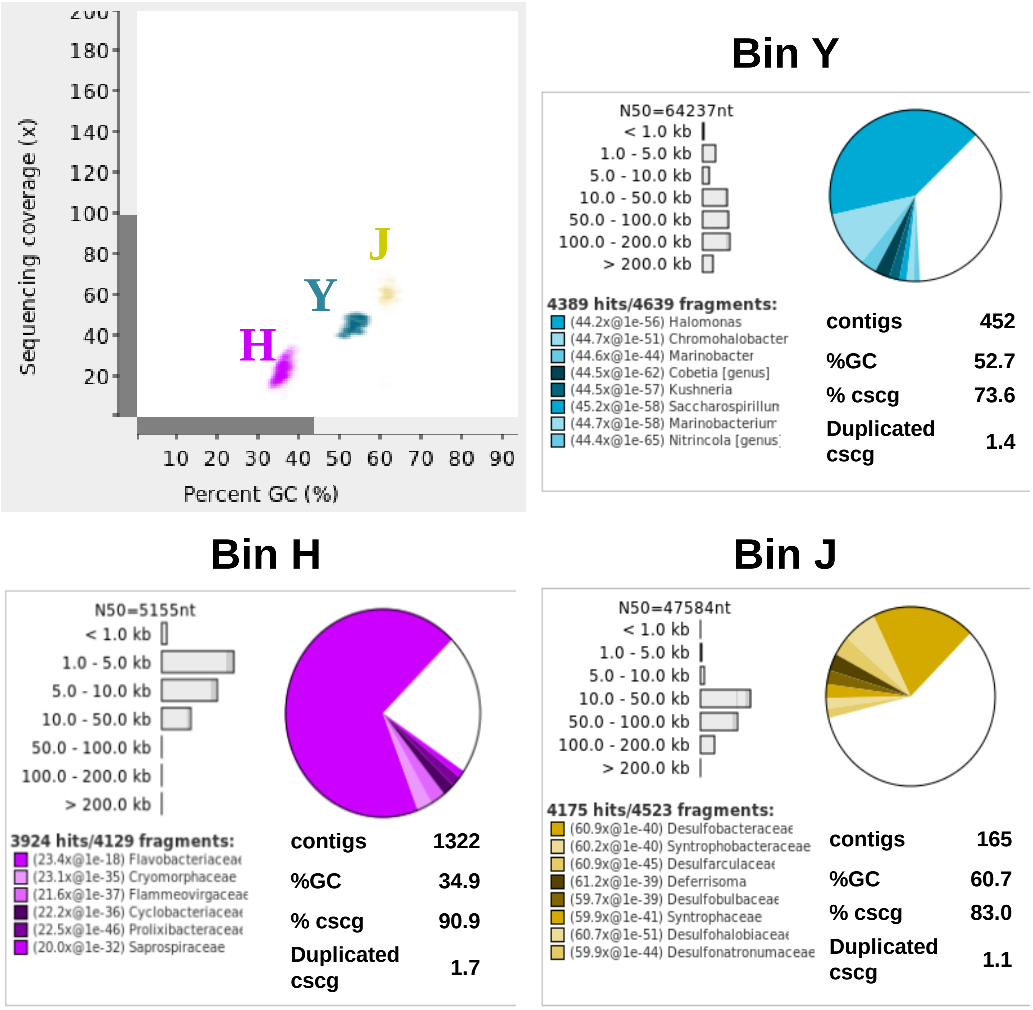

Supplementary Figure S5. Automatic binning of Socompa contigs performed by Metawatt 3.2. Selected and manually polished high quality bins are shown, including the affiliation of the encoded proteins for each bin.

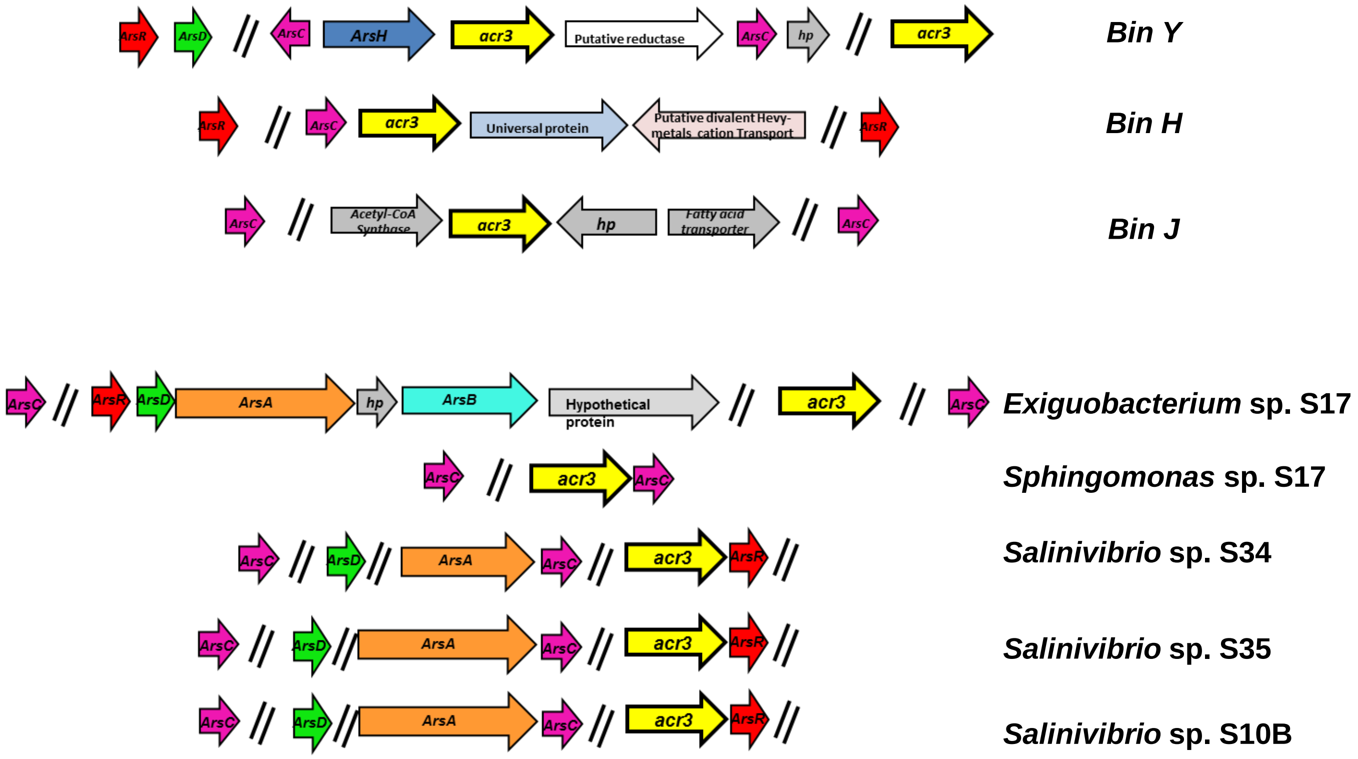

Supplementary Figure S6. Arsenic resistance genes in binned sequences from Socompa metagenome and in strains previously isolated from the stromatolites [21,68,70].
